# Künstliche Intelligenz in der Mammadiagnostik: Anwendungsgebiete aus klinischer Perspektive

**DOI:** 10.1007/s00117-020-00802-2

**Published:** 2021-01-28

**Authors:** Pascal A. T. Baltzer

**Affiliations:** Universitätsklinik für Radiologie und Nuklearmedizin, allgemeines Krankenhaus der Medizinischen Universität Wien, Währinger Gürtel 18–20, 1090 Wien, Österreich

**Keywords:** Brustkrebs, Früherkennung, Mammographie, Software, Personalisierte Medizin, Breast cancer, Early diagnosis, Mammography, Software, Precision medicine

## Abstract

**Klinisches/methodisches Problem:**

Bei der Mammadiagnostik gilt es, klinische sowie multimodal bildgebende Informationen mit perkutanen und operativen Eingriffen zu koordinieren. Aus dieser Komplexität entsteht eine Reihe von Problemen: übersehene Karzinome, Überdiagnose, falsch-positive Befunde, unnötige weiterführende Bildgebung, Biopsien und Operationen.

**Radiologische Standardverfahren:**

Folgende Untersuchungsverfahren werden in der Mammadiagnostik eingesetzt: Röntgenmammographie, Tomosynthese, kontrastangehobene Mammographie, (multiparametrischer) Ultraschall, Magnetresonanztomographie, Computertomographie, nuklearmedizinische Verfahren sowie deren Hybridvarianten.

**Methodische Innovationen:**

Künstliche Intelligenz (KI) verspricht Abhilfe bei praktisch allen Problemen der Mammadiagnostik. Potenziell lassen sich Fehlbefunde vermeiden, bildgebende Verfahren effizienter einsetzen und möglicherweise auch biologische Phänotypen von Mammakarzinomen definieren.

**Leistungsfähigkeit:**

Auf KI basierende Software wird für zahlreiche Anwendungen entwickelt. Am weitesten fortgeschritten sind Systeme für das Screening mittels Mammographie. Probleme sind monozentrische sowie kurzfristig am finanziellen Erfolg orientierte Ansätze.

**Bewertung:**

Künstliche Intelligenz (KI) verspricht eine Verbesserung der Mammadiagnostik. Durch die Vereinfachung von Abläufen, die Reduktion monotoner und ergebnisloser Tätigkeiten und den Hinweis auf mögliche Fehler ist eine Beschleunigung von dann weitgehend fehlerfreien Abläufen denkbar.

**Empfehlung für die Praxis:**

In diesem Beitrag werden die Anforderungen der Mammadiagnostik und mögliche Einsatzgebiete der der KI beleuchtet. Je nach Definition gibt es bereits praktisch anwendbare Softwaretools für die Mammadiagnostik. Globale Lösungen stehen allerdings noch aus.

## Abstract (en)

**Clinical/methodological issue:**

Central to breast imaging is the coordination of clinical and multimodal imaging information with percutaneous image-guided biopsies and surgical procedures. A wide range of problems arise due to this complexity: missed cancers, overdiagnosis, false-positive findings, unnecessary further imaging, biopsies and surgeries.

**Standard radiological methods:**

Breast imaging comprises the following diagnostic tests: mammography, tomosynthesis, contrast-enhanced mammography, (multiparametric) ultrasound, magnetic resonance imaging, computed tomography, nuclear medicine derived imaging and hybrid methods.

**Methodological innovations:**

Artificial intelligence (AI) promises to alleviate practically all these problems of breast imaging. AI has the potential to avoid missed cancers and false-positive findings. Furthermore, it could guide an efficient use of imaging methods and it may potentially be used to define biological phenotypes of breast cancer.

**Performance:**

AI-based software is being developed for various applications. Most developed are systems that support mammography screening. Problems are monocentric approaches and the focus on short-term financial success.

**Achievements:**

AI promises to improve breast imaging by simplifying and speeding up the workflow, by reducing monotonous tasks and by pointing out problems. This is likely to set free physician capacities that could be invested in improved communication with patients and interdisciplinary colleagues.

**Practical recommendations:**

The present article mainly addresses clinical needs in breast imaging, pointing out potential areas of use for artificial intelligence. Depending on the definition, a wide array of helpful software tools for breast imaging are already available. Global solutions, however, are still missing.

Der Arbeitsablauf in der Mammadiagnostik lässt sich in 3 Bereiche aufteilen: Früherkennung (Screening), Abklärung von auffälligen Screeningbefunden (Assessment) bzw. von symptomatischen Patientinnen (der Arbeitsablauf ist ähnlich) sowie das prätherapeutische Management bekannter Karzinome inklusive der Beurteilung des Ansprechens auf neoadjuvante Therapien und der präoperativen Markierung bildgebender Befunde (Abb. 1).

**Figure 1:**
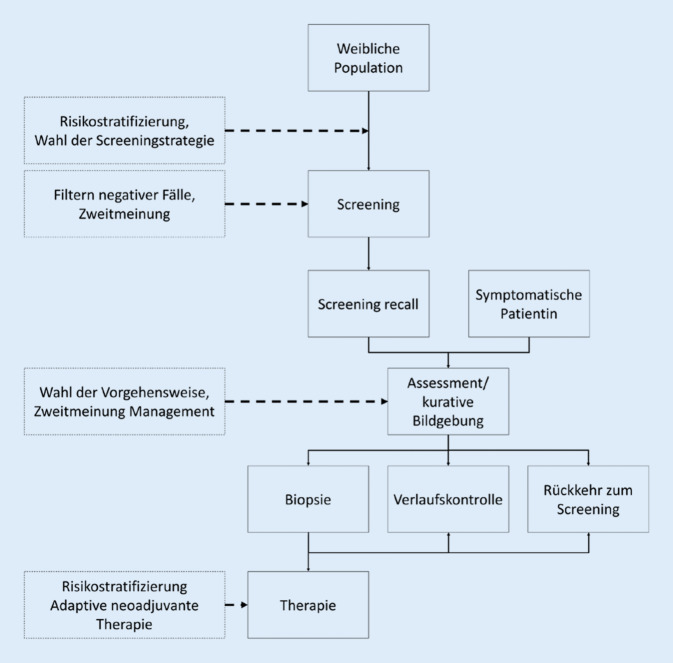

## Früherkennung/Screening

Internationale Fachgesellschaften empfehlen die Röntgenmammographie zur Früherkennung (Sekundärprävention) von Brustkrebs [1]. Wenn auch die Mammographie ein einfaches, relativ kostengünstiges und vor allem robustes Verfahren mit lange etablierten Qualitätssicherungsmaßnahmen darstellt, sind die Schwächen dieser Herangehensweise bekannt: Das Risiko für Brustkrebs ist in der weiblichen Bevölkerung nicht gleichverteilt. Während Frauen mit bekannter Genmutation mit sehr hoher Wahrscheinlichkeit im Laufe ihres Lebens an Brustkrebs erkranken, trifft diese Erkrankung andere Frauen niemals. Die Bestimmung des Brustkrebsrisikos ist Gegenstand interdisziplinärer Forschung und wird hier nicht im Detail behandelt. Als mutmaßlich wichtigster unabhängiger Risikofaktor gilt die mammographische Brustdichte. Sie lässt sich sowohl subjektiv kategorial nach dem BI-RADS-Lexikon als auch quantitativ bestimmen. Unabhängig von der Herangehensweise ist eine erhöhte Brustdichte mit einem erhöhten Risiko, an Brustkrebs zu erkranken, assoziiert [2, 3]. Zudem beeinflusst die Brustdichte auch die Sensitivität der Mammographie: Je dichter die Brust, desto weniger wahrscheinlich werden gerade kleine Tumoren mittels Mammographie detektiert [4]. Ein Surrogatmarker für die Anzahl verpasster, biologisch aggressiver Tumoren spiegelt sich in der Rate von Intervallkarzinomen wider [4, 5]. Intervallkarzinome werden im Screening-Intervall klinisch apparent. Dabei stellt die Brustdichte einen breit verfügbaren Biomarker dar, welcher für eine individuell adaptierte Screeningstrategie herangezogen werden kann. Frauen mit erhöhter Brustdichte benötigen zusätzliche Tests zur Optimierung der Tumordetektionsraten [6]. Das vielversprechendste Verfahren dafür ist die Magnetresonanztomographie (MRT) der Mamma. Eine prospektiv-randomisierte Studie an Frauen mit quantitativ erfasster extrem hoher Brustdichte konnte überzeugende Zahlen zur Reduktion der Intervallkarzinome und damit biologisch signifikanter Befunde belegen [5]. Dieser Ansatz wurde zudem als kosteneffektiv bewertet [7]. Gemessen an den Intervallkarzinomraten wäre ein Einsatz der MRT auch bei mäßig erhöhter Brustdichte erwägenswert [4]. Bei suffizienter Einbindung in RIS/PACS-Systeme könnte der Prozess einer individualisierten Auswahl passender Untersuchungen und Untersuchungsintervalle in der Screeningsituation mittels künstlicher Intelligenz (KI) weitgehend automatisiert werden ([8]; Abb. 1).

Ein ungelöstes Problem des Brustkrebs-Screenings ist die sog. Überdiagnose. Der Begriff beschreibt die Diagnose und Therapie von wenig aggressiven, die Prognose der Patientinnen nicht beeinflussenden Karzinomen [9]. Allein am histologischen bzw. immunhistochemischen Tumortyp lässt sich ein Brustkrebs derzeit nicht verlässlich als klinisch relevant oder irrelevant bewerten. Deshalb bleibt Überdiagnose ein klinisch abstrakter Begriff, und die Raten *überdiagnostizierter* Karzinome lassen sich lediglich epidemiologisch durch persistierend die Hintergrundinzidenz übersteigende Tumordetektionsraten abschätzen. Das Problem der Überdiagnose ist nicht die Diagnose selbst, sondern die daraus folgende Therapie [9]. Diese folgt Leitlinien, welche neuen Erkenntnissen erst Rechnung tragen müssen. Der Begriff Überdiagnose nebst der damit verbundenen Kritik am Mammographie-Screening ist somit irreführend und weist auf ein überholtes Verständnis von Medizinethik und Wissenschaft hin: Vielmehr ist die Diagnose solcher Tumoren notwendig für die Entwicklung verbesserter diagnostischer und therapeutischer Strategien. Die Diskussion dieses Begriffs verschleiert auch das wirkliche Problem der Brustkrebsdiagnostik: die *Unterdiagnose* klinisch signifikanter Mammakarzinome, direkt messbar an Intervallkarzinomen und der trotz Verbesserung der diagnostischen und therapeutischen Verfahren weiterhin sehr hohen Sterblichkeit an Brustkrebs [10]. Die breite Verfügbarkeit funktioneller bildgebender Biomarker hat ein großes Potenzial, mit Hilfe individualisierter Phänotypisierung von Brustkrebs Therapieentscheidungen zu unterstützen. Zur Integration dieser Informationsfülle werden Verfahren der KI mit hoher Wahrscheinlichkeit erforderlich ([11, 12]; Abb. 1).

## Abklärung von Screening-Recalls und symptomatischen Patientinnen

**Figure 2:**
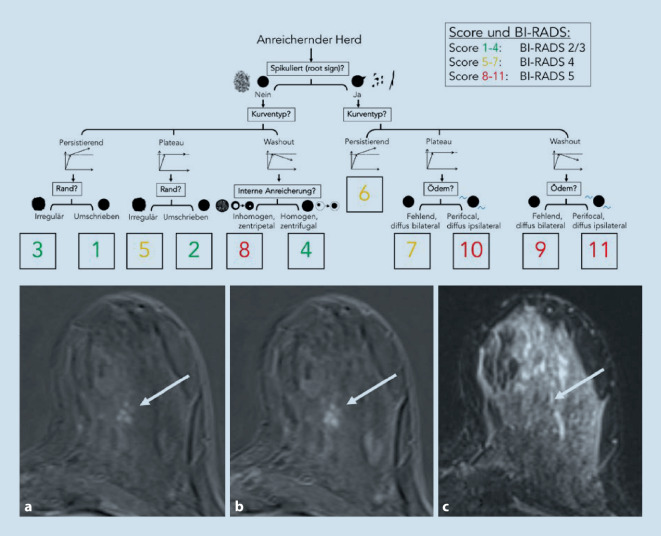

**Figure 3:**
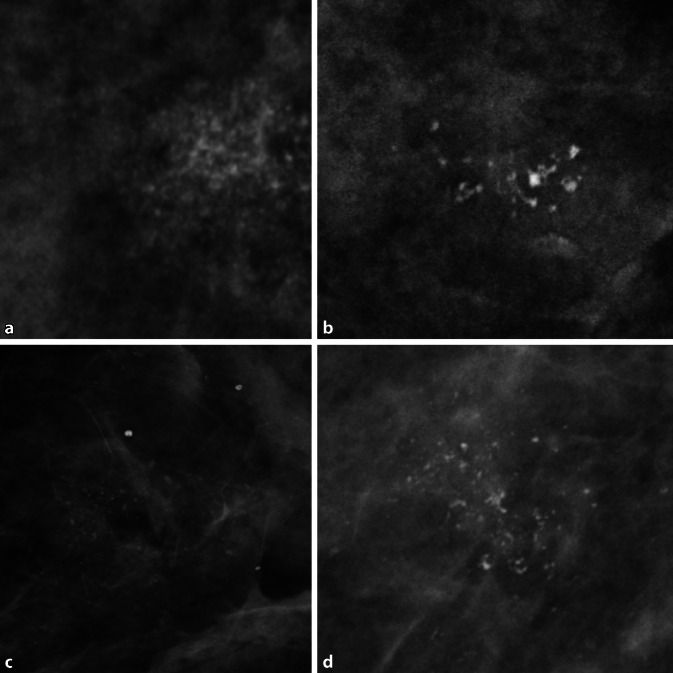

Auch aufgrund erheblicher Unterschiede bei der Vergütung medizinischer Leistungen zwischen verschiedenen Ländern erfolgt die Abklärung auffälliger Befunde weniger einheitlich als das Screening. Neben gezielten mammographischen Aufnahmen bieten sich vor einer bildgezielten Biopsie weitere diagnostische Verfahren wie Ultraschall, Kontrastmittel-Mammographie oder MRT an ([13]; Abb. 1). Die Wahl der weiterführenden Diagnostik obliegt den jeweiligen RadiologInnen, welche die individuellen Befunde der Patientin für ihre Managemententscheidung in Betracht ziehen müssen. Diese ist im Fall bildgebend oder klinisch eindeutig lokalisierbarer Auffälligkeiten einfach: Internationale Richtlinien empfehlen die perkutane Biopsie zur sicheren Diagnose [14–16]. Weiterführende Bildgebung wird vorrangig zur Planung von Biopsien eingesetzt. Diffuse, schlecht lokalisierbare Prozesse lassen sich nicht direkt biopsieren [17]. Das gilt mit Einschränkung auch für ausgedehnte, nichtsolide Prozesse, wie Verkalkungen. Die Biopsie eines Teils der Verkalkung mag einen gutartigen Befund ergeben, schließt jedoch Malignität in den Augen erfahrener Kliniker nicht ausreichend aus („sampling error“). Eine Möglichkeit stellen weiterführende bildgebende Verfahren dar [17–20]. Während die Magnetresonanztomographie mit Sicherheit das akkurateste Verfahren in diesem Setting ist (nur die MRT erreicht aufgrund ihres hohen negativen Vorhersagewerts eine ausreichende Sicherheit, um einen Verzicht auf Biopsie zu rechtfertigen [21]), stellt sie einen zusätzlichen Aufwand dar und kann die Situation durch potenzielle falsch-positive Befunde verkomplizieren [22, 23]. Während der ökonomische Aufwand stark von den politisch akzeptierten Kosten der alternativen Verfahrensweisen abhängt (für die MRT der Mamma Faktor 10 und höher in Ländern mit vergleichbarem sozioökonomischem Status), ist der organisatorische Aufwand einfacher zu bemessen. Selbst bei guter Verfügbarkeit von Magnetresonanztomographien ist eine Verzögerung des Managements von zumindest Stunden, wahrscheinlicher Tagen zu erwarten. Gerade deshalb erfreut sich die Kontrastmittel-Mammographie trotz klar unterlegener diagnostischer Aussagekraft großer Beliebtheit [24, 25]. Nicht zu unterschätzen sind auch multiparametrische Ultraschallverfahren [26]. Alle lassen sich noch vor Ort mit geringem Zeitverlust einsetzen. Hier ergeben sich mehrere Einsatzgebiete für KI-basierte Lösungen [27–29]. Zum einen könnte die individuelle Patienteninformation zusammen mit bisher erhobenen bildgebenden Befunden in einer KI-basierten Managementempfehlung münden [30]. Dank *Machine Learning* lassen sich aus standardisierten Routinekriterien objektivierbare klinische Entscheidungsregeln erstellen, zuletzt überzeugend als der *Kaiser-Score* für die MRT der Mamma. Diese klinische Entscheidungsregel (http://www.meduniwien.ac.at/kaiser-score/) ermöglicht sichere Diagnosen von kontrastmittelaffinen Läsionen und hat ihren (Mehr)wert in multiplen klinischen Szenarien gezeigt (Abb. 2; [31–33]). Möglicherweise noch eleganter ist der Einsatz KI-basierter Systeme zur automatisierten Evaluation bildgebender Veränderungen im Sinne einer Zweitmeinung mit dem Ergebnis einer objektiven und möglicherweise verbesserten Einschätzung des Karzinomrisikos (Abb. 3 und 4). Ein KI-Algorithmus kann dabei eine Wahrscheinlichkeit für das Vorliegen der gesuchten Pathologie (z. B. invasiver Brustkrebs) ausgeben. Befunde mit sehr niedrigem Risiko könnten gefahrlos verlaufskontrolliert werden [34, 35]. Der Einsatz von KI kann damit potenziell unnötige weiterführende Bildgebung und Eingriffe vermeiden.

**Figure 4:**
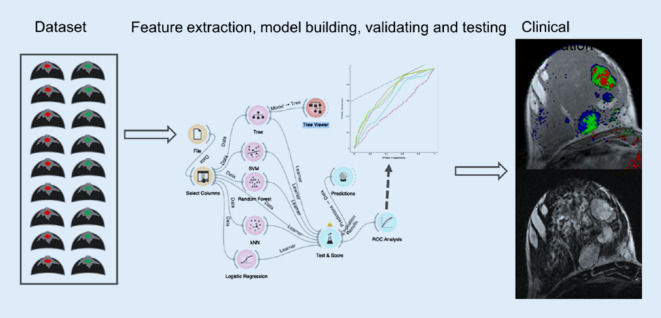

## Management bekannter Karzinome

Das Management histologisch gesicherter Karzinome stellt einen dritten Schwerpunkt der Bildgebung nebst bildgezielter Interventionen dar (Abb. 1). Der immunhistochemisch am Biopsiepräparat determinierte Tumortyp bestimmt weitgehend die therapeutische Herangehensweise: sofortige Operation oder neoadjuvante medikamentöse Behandlung [36]. Spezielle Entscheidungen werden jedoch durch den bildgebenden Befund entscheidend beeinflusst. Das Lokalstaging ist eine Domäne der Bildgebung und bestimmt die Möglichkeit einer brusterhaltenden Therapie. Auch die Lymphknotendiagnostik und ggf. gezielte Biopsie von Lymphknoten hat in den letzten Jahren an Bedeutung gewonnen [16]. Im Fall neoadjuvanter Therapien werden bildgebende Verfahren zur Beurteilung des Therapieansprechens angewendet [37]. Eine akkurate Diagnose der Vitalität residueller Tumoranteile oder ein frühzeitig im Therapieverlauf mittels bildgebender Marker zu diagnostizierendes Therapieversagen wären für die Adaption von Therapiestrategien äußerst wünschenswert [38]. Jede Operation, auch solche nach neoadjuvanter Therapie, erfordert zumindest bei diffusen, multifokalen oder klinisch nichtpalpablen Befunden eine operative Markierung der Befunde, anhand derer sich ChirurgInnen in situ orientieren können [16]. Gerade in diesem Bereich fehlt es an klaren Standards aufgrund der starken Variabilität der individuellen Fälle [39]. KI-basierte Ansätze könnten hier Abläufe entscheidend optimieren [8, 27].

## Einsatzgebiete der künstlichen Intelligenz

### Radiologische Herausforderungen

Die obigen Ausführungen und Abb. 1 zeigen verschiedene attraktive Ansatzpunkte für die Anwendung von künstlicher Intelligenz in der Mammadiagnostik auf. Computertechnik hat in der heutigen Radiologie einen zentralen Stellenwert, Befunde werden dank Digitalisierung nebst Spracherkennung möglichst unmittelbar nach der Untersuchung erwartet. [8]. Die Verbesserung der bildgebenden Gerätetechnik hat zusammen mit effizienteren digitalen Arbeitsplätzen in den letzten Jahrzehnten zu einer ausgeprägten Verdichtung der radiologischen Arbeit geführt [40, 41]. Radiologische Leistungen im Jahr 2020 werden insbesondere außerhalb spezialisierter akademischer Einrichtungen im Akkord erarbeitet. Für einen der zentralen Punkte der Diagnostik, nämlich die Befundvermittlung an Patienten und Zuweiser bleibt wenig Zeit [42]. Genau diese Probleme lassen sich potenziell durch KI-Verfahren lösen [8]. Der weit gefasste Begriff umschließt dabei Anwendungen zur Fehlererkennung, zur Identifizierung von unauffälligen Befunden sowie zur Vermeidung von Fehlern. All das lässt sich auch bereits im Vorfeld bei der Stellung der Untersuchungsindikation und Bildakquisition anwenden. Der rein digitale Arbeitsplatz erlaubt eine flexible Einbindung von softwarebasierten Lösungen der genannten Probleme.

### Erforschung der KI

Künstliche Intelligenz ist generell ein unscharfer Ausdruck, welcher in den vergangenen Jahrzehnten seit seiner Prägung vielfach inflationär eingesetzt wurde [8, 27, 28]. Gerade in der Radiologie wurde die Rolle der KI oftmals grotesk überschätzt [8]. Künstliche Intelligenz impliziert Autonomität und Kreativität. Beides trifft auf die heute verfügbaren Verfahren, welche von statistischer Klassifikation bis hin zu Deep Learning reichen, nicht zu. Die durch Fortschritte in der Computertechnik erreichten Steigerungen der Rechenkapazität gestatten heute die Anwendung erheblich komplexerer Modelle bzw. eine zeiteffiziente Anwendung derselben [29]. Das ermöglicht zwar die Lösung komplexer, nichtlinearer Probleme, erfordert aber entsprechend robuste Lösungen. Fehlende Robustheit oder Generalisierbarkeit von Algorithmen der KI stellt die Achillesferse derselben dar. Sie hängt kritisch von der verfügbaren Datenmenge und der erreichbaren Standardisierung ab [8]. Der klassische translationale Ansatz von akademischer Forschung, deren Ergebnisse durch Unternehmen kommerzialisiert werden, ist hier einerseits einfacher, andererseits schwerer zu realisieren. Einfacher, weil die Aufnahmen bildgebender Verfahren direkt als Daten verstanden werden können [43]. Schwerer, weil die Forschung auf dem Gebiet große Datenmengen zielorientiert filtert, also direkten Hypothesen folgt. Ohne vorherige Standardisierung der Datenarchive ist der Erfolg dieses Ablaufs jedoch limitiert [44]. Der klassische Ansatz von kleinen Forschungsgruppen, welche mit beschränkten lokalen Datenbanken Ergebnisse generieren und publizieren, ist bei Studien zur künstlichen Intelligenz gefährlich. Die Vielzahl der verfügbaren Daten, welche miteinander assoziiert werden, impliziert eine hohe Rate falsch-positiver Forschungsergebnisse. Insbesondere bei limitierter Studienqualität können die echten (richtig-positiven) Ergebnisse verschleiert werden [44, 45]. Man kann Forschung auf dem Gebiet der KI mit industriellem Fischfang in trübem Wasser vergleichen. Der Forscher hofft auf einen wertvollen Fund, muss diesen allerdings unter Unmengen irrelevanter Ergebnisse identifizieren. Ohne auf die Datenschutzproblematik einzugehen, ist auch die Zusammenarbeit mit der Wirtschaft in diesem Fall nicht gefahrlos. Die zumindest auf mittelfristigen finanziellen Erfolg ausgerichtete Strategie von Unternehmen impliziert eine Vernachlässigung der Sorgfaltspflicht, mangelhafte Kritik und Überbewertung von ökonomischem Potenzial [46]. Wie bereits für genomische und proteomische Forschung angeregt, stellen nur große interdisziplinäre Kollaborationen zwischen Forschern und Unternehmen einen nachhaltigen Ansatz dar [45].

### Integration in den klinischen Alltag

Die Erstellung eines KI-Tools impliziert noch keine problemfreie Anwendung. Wie kürzlich ganz ausgezeichnet in einem dedizierten Artikel zusammengefasst, erfordert die produktive Anwendung von KI-Lösungen ganz grundlegende organisatorische und administrative Schritte [8]. Der beste Algorithmus zur Klassifikation von Herdbefunden ist beispielsweise hilflos, wenn eine uneinheitliche Serienbezeichnung (z. B. nach einem Softwareupdate des Geräteherstellers) die Identifikation des zu analysierenden Bildmaterials nicht ermöglicht. Gerade bei KI-Lösungen in größerem Maßstab lässt sich der Fehler dann nicht einfach lokalisieren und die präzise Definition des Workflows und der Fehlermeldung sind entscheidend für die Lösung des Problems. Natürlich ließe sich dies durch eine weitere KI-Lösung oder Vernetzung der Techniken beheben, was nur die aktuelle Problematik widerspiegelt: Ein Großteil der Forscher und Unternehmen arbeiten in einer Art Goldrausch an eigenen und originellen Lösungen, und wie schon bei anderen Industriezweigen ist die Definition von KI-spezifischen Standards eine der großen Herausforderungen für das ganze Gebiet [8, 27, 46].

## Fazit für die Praxis

- Verfahren der künstlichen Intelligenz versprechen eine nachhaltige Verbesserung der Mammadiagnostik durch Vereinfachung von Abläufen, Reduktion monotoner und ergebnisloser Tätigkeiten und den Hinweis auf mögliche Fehler.
- Die dadurch freigesetzten ärztlichen Kapazitäten könnten in eine verbesserte Kommunikation mit PatientInnen und interdisziplinären KollegInnen im Sinne einer personalisierten Medizin eingesetzt werden.
- Der vorliegende Text hat mit dem Ziel einer kommerziell neutralen Präsentation absichtlich bereits erhältliche Produkte (zumeist für die Anwendung im Screening nebst quantitativer Brustdichtemessung) von der Darstellung ausgeschlossen. Allen derzeit verfügbaren Lösungen gemein ist das Fehlen eines echten integrativen Ansatzes.
- KI-basierte Tools benötigen für eine zielgerichtete Anwendung eine genaue Definition der lokalen Bedürfnisse und Gegebenheiten und müssen auf diese zugeschnitten werden.
- Echte, überregionale und fächerübergreifende KI-Lösungen für die Senologie sind zwar bereits abzusehen, jedoch in den nächsten Jahren noch nicht zu erwarten.
